# Modeling Alzheimer’s Disease in Mouse without Mutant Protein Overexpression: Cooperative and Independent Effects of Aβ and Tau

**DOI:** 10.1371/journal.pone.0080706

**Published:** 2013-11-20

**Authors:** Qinxi Guo, Hongmei Li, Allysa L. Cole, Ji-Yeun Hur, Yueming Li, Hui Zheng

**Affiliations:** 1 Huffington Center on Aging, Baylor College of Medicine, Houston, Texas, United States of America; 2 Molecular Pharmacology and Chemistry Program, Memorial Sloan-Kettering Cancer Center, New York, New York, United States of America; Cleveland Clnic Foundation, United States of America

## Abstract

**Background:**

Alzheimer’s disease (AD), the most common cause of dementia in the elderly, has two pathological hallmarks: Aβ plaques and aggregation of hyperphosphorylated tau (p-tau). Aβ is a cleavage product of Amyloid Precursor Protein (APP). Presenilin 1 (PS1) and presenilin 2 (PS2) are the catalytic subunit of γ-secretase, which cleaves APP and mediates Aβ production. Genetic mutations in *APP*, PSEN1 or *PSEN2* can lead to early onset of familial AD (FAD). Although mutations in the tau encoding gene MAPT leads to a subtype of frontotemporal dementia and these mutations have been used to model AD tauopathy, no MAPT mutations have been found to be associated with AD.

**Results:**

To model AD pathophysiology in mice without the gross overexpression of mutant transgenes, we created a humanized AD mouse model by crossing the *APP* and *PSEN1* FAD knock-in mice with the htau mice which express wildtype human *MAPT* genomic DNA on mouse *MAPT* null background (APP/PS1/htau). The APP/PS1/htau mice displayed mild, age-dependent, Aβ plaques and tau hyperphosphorylation, thus successfully recapitulating the late-onset AD pathological hallmarks. Selected biochemical analyses, including p-tau western blot, γ-secretase activity assay, and Aβ ELISA, were performed to study the interaction between Aβ and p-tau. Subsequent behavioral studies revealed that the APP/PS1/htau mice showed reduced mobility in old ages and exaggerated fear response. Genetic analysis suggested that the fear phenotype is due to a synergic interaction between Aβ and p-tau, and it can be completely abolished by tau deletion.

**Conclusion:**

The APP/PS1/htau model represents a valuable and disease-relevant late-onset pre-clinical AD animal model because it incorporates human AD genetics without mutant protein overexpression. Analysis of the mice revealed both cooperative and independent effects of Aβ and p-tau.

## Introduction

Alzheimer’s disease (AD) is the most common form of age-related neurodegenerative disorder characterized by the presence of extracellular amyloid plaques consisting of β-amyloid peptides (Aβ) and intracellular neurofibrillary tangles (NFT) composed of hyperphosphorylated tau (p-tau) protein in diseased brains. Although most AD cases occur after 65 years of age, a subset of patients develop AD clinical symptoms much younger due to autosomal dominant mutations in *APP*, *PSEN1* or *PSEN2* [1]. These familial AD (FAD) containing mutant proteins alter the production of Aβ, leading to accelerated amyloid pathology. Tau is encoded by the *MAPT* gene. Interestingly, although *MAPT* mutations are linked with other types of human dementia, such as frontotemporal dementia (FTD) and corticobasal degeneration (CBD), no *MAPT* mutation is found to be associated with AD [2].

A pre-clinical animal model of AD that can faithfully mimic the human conditions is crucial to understand the disease mechanism and to test for novel therapeutic strategies. So far, many transgenic mouse models expressing mutant *APP*, *PSEN1* and/or *MAPT* under the control of exogenous promoters have been generated and have provided valuable knowledge regarding AD pathogenesis. However, in order to accelerate pathological development, transgenic mouse models generally overexpress mutant proteins at high levels. Considering the crucial and various physiological functions of APP, PS1 and tau in the central nervous system, it begs the question whether the biochemical and functional defects observed in AD transgenic mice are due to the pathological lesions or because of mutant protein overexpression [3].

To avoid the complications of transgenic protein overexpression and to build a more physiologically relevant model, AD knock-in models were generated by introducing human *APP* and/or *PSEN1* FAD mutations and humanized Aβ to the endogenous mouse gene [3-8]. Compared to traditional transgenic models, knock-in mice have unique advantages. Because the knock-in allele is under the native promoter control, the protein expression remains at physiological levels, and the temporal and spatial expression patterns are not disturbed. Additionally, in contrast to the transgenic models in which the existence of mouse proteins may complicate the phenotypes, the mouse gene products are replaced with the humanized mutant proteins in knock-in models. Nevertheless, despite the expression of human Aβ, no tau abnormality has been reported in the APP knock-in models. 

Andorfer et al. discovered that endogenous mouse tau may be protective against pathological aggregations [9], and in the same study a humanized tau mouse model was generated to express a full-length wildtype human *MAPT* genomic fragment along with its regulatory sequences on a mouse *MAPT* knock-out background (htau). The htau mice express all six human tau isoforms and show age-dependent tau hyperphosphorylation and aggregation.

Here we crossed the APP/PS1 knock-in mice with the htau mice to create a new AD model that recapitulates human AD genetics, FAD mutations with wildtype human tau, in the absence of transgene overexpression. We systematically evaluated the biochemical, histological and behavioral features of the mice, and the findings provided novel insights into mechanisms of AD pathogenesis.

##  Materials and Methods

### Ethics Statement

All experimental protocols were reviewed and approved by the Baylor College of Medicine Institutional Animal Care and Use Committee (IACUC) and performed in compliance with animal warfare guidelines of National Institutes of Health (NIH).

### Mouse husbandry and breeding

**Figure 1:**
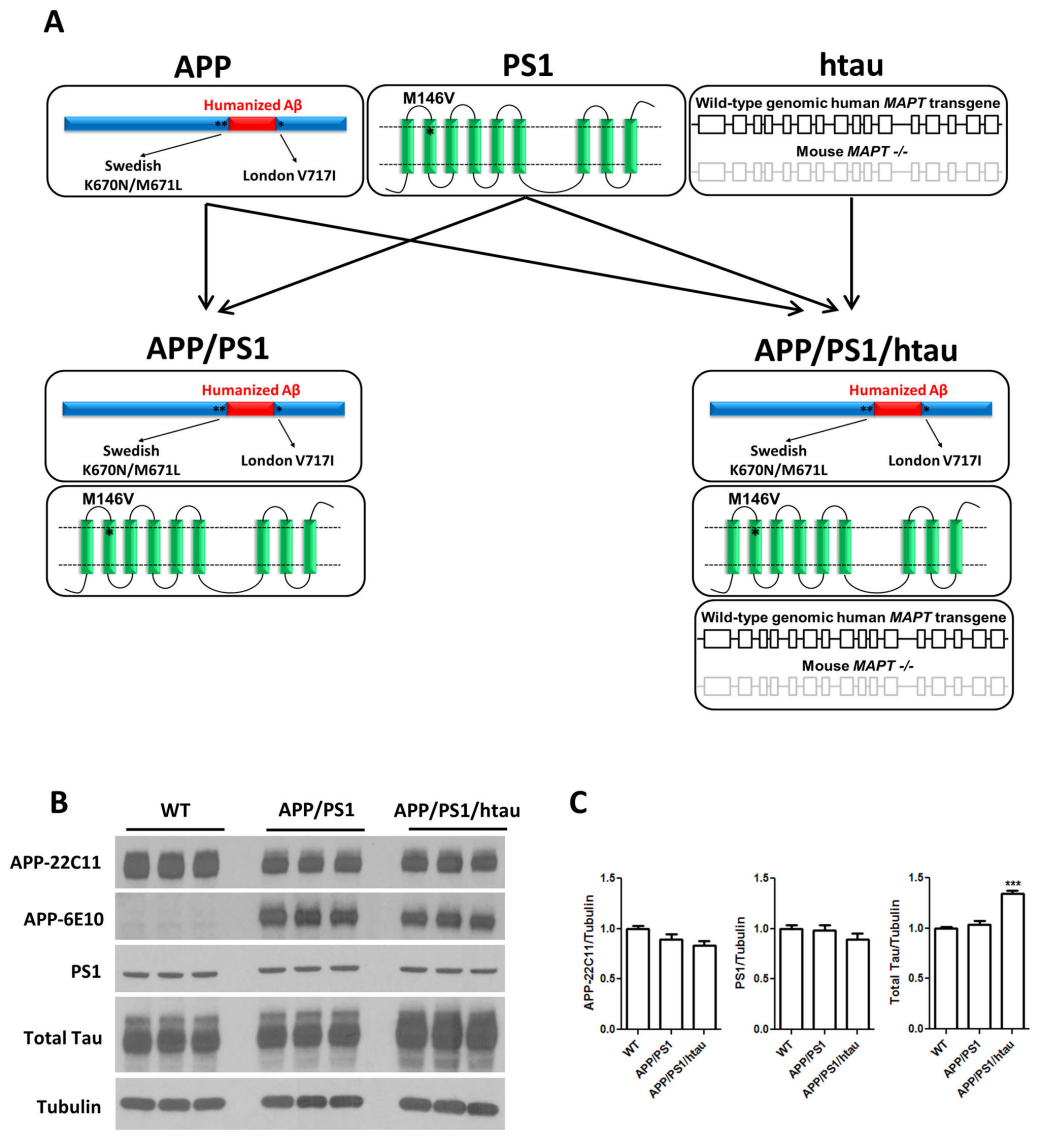
Knock-in mouse models show physiological levels of protein expression. A. Generation of the APP/PS1/htau mouse model with knock-in APP and PS1 FAD mutations as well as humanized Aβ and wild-type human MAPT gene. B. Western blot analysis and quantification using APP (22C11), APP (6E10) recognizing human Aβ region, PS1, total tau, and γ-tubulin antibodies against hippocampal lysates from WT, APP/PS1 and APP/PS1/htau animals at 16 months of age. C. Statistics on band intensities of APP (22C11)/tubulin, PS1/tubulin, and total tau/tubulin, demonstrating no overexpression of APP and PS1, existence of humanized Aβ in the APP/PS1 and APP/PS1/htau models, and mild overexpression of total tau in the APP/PS1/htau model. *** p<0.001, one-way ANOVA with Newman-Keuls multiple comparison test.

All mice used for this study have been backcrossed for at least 6 generations onto the C57BL/6 background. The APP homozygous knock-in mice expressing the Swedish/London mutations and humanized Aβ sequence (APP) [8] and PS1 homozygous knock-in mice containing the M146V mutation (PS1) [5] were bred together to generate the homozygous APP/PS1 double knock-in animals as reported earlier [10]. The APP/PS1 animals were subsequently crossed to htau mice to generate the APP/PS1/htau mice that are homozygous for APP and PS1 but hemizygous for human genomic *MAPT* on mouse tau knockout background (Figure 1A).

### Antibodies

22C11 (Millipore, mouse monoclonal, 1:1000) recognizing both human and mouse APP, and 6E10 (Covance, mouse monoclonal, 1:1000) which is specific for human Aβ region, were used for APP western blot. PS1-N antibody (rabbit polyclonal, 1:1000, previously described [11]) and BACE antibody (Cell Signaling # 5606, rabbit monoclonal, 1:1000) were used to detect PS1 and BACE expression on western blot, respectively. β-amyloid antibody (Cell Signaling #2454, rabbit polyclonal, 1:2000) and homemade Aβ antibody (U6950, rabbit polyclonal, 1:2000) were used for the Aβ plaque immunofluorescence and immunohistochemistry staining. The phosphorylated tau antibodies used in this study include CP13 (mouse monoclonal, 1:500 to 1:5000), PHF-1 (mouse monoclonal, 1:500 to 1:5000), which were generous gifts from Dr. Peter Davis, AT180 (Thermo Scientific, mouse monoclonal, 1:500), and AT270 (Thermo Scientific, mouse monoclonal, 1:500). The total tau antibody used in western blots is anti-tau (DAKO A0024, rabbit polyclonal, 1:20000). The anti-γ-tubulin antibody (mouse monoclonal, 1:10000) was purchased from Sigma.

### Immunofluorescence and immunohistochemistry

TBS buffer was used in all procedures in lieu of PBS to avoid complications for p-tau staining. Anesthetized animals were perfused with 4% PFA in sodium tetraborate decahydrate (Borax) and brains were dissected out for overnight post-fixation in 4% PFA in Borax. Brains were incubated in 30% sucrose solution in TBS overnight, sectioned on a sliding microtome (Leica SM2000R) at the thickness of 30 µm and preserved in cryoprotectant solution (1.59g NaH_2_PO_4_H_2_O, 5.47g Na_2_HPO_4_, 9.0g NaCl, 300g Sucrose, 10.0g Polyvinylpyrrolidone, 300 mL Ethylene Glycol, fill to 1L with ddH2O). For antibody staining, floating brain sections were rinsed with TBS three times, incubated with blocking solution (TBS with 3% BSA, 2% goat or donkey serum and 0.4% Triton X-100) for at least 1 hour, incubated with primary antibody solution (primary antibody diluted in blocking solution) at 4°C overnight, rinsed with TBS for three times, incubated with secondary antibody solution for 2 hours, and then rinsed with TBS three times. For fluorescence staining, brain sections were then mounted on gelatin coated slides with mounting medium containing DAPI (Vector Lab). In case of DAB chemical staining, the Elite ABC staining kit (Vector Lab) was used according to the user manual. 

### Western Blot

Mouse brains were dissected out in cold TBS containing cocktail protease and phosphatase inhibitors (Roche). Brain samples were homogenized in cold 50mM Tris-HCl pH7.0 buffer with 150mM NaCl, 1mM EDTA, 1% NP-40 and cocktail protease and phosphatase inhibitors (Roche). Protein concentration of each sample was quantified by DC™ Protein Assay (Bio-Rad) and subjected to SDS-PAGE followed by western blot analysis.

### *In vitro* γ-secretase Activity Assay and Aβ ELISA

The γ-secretase activity assay using a recombinant substrate Sb4 for the γ-secretase cleavage, which was expressed from bacterial culture, was performed according to previous reports [12-15]. For Aβ ELISA, mouse brains were homogenized in TBS containing 1% Triton X-100. Aβ levels of the supernatant after centrifugation at 20,000g was measured using the Invitrogen human Aβ40 ELISA kit (#KHB3482, Frederick, MD), and the Invitrogen human Aβ42 US ELISA kit (#KHB3544, Frederick, MD) following the manufacturer’s instructions.

### Mouse Behavior Tests

*Morris Water Maze (MWM*) The water maze is a white plastic pool (130cm in diameter) filled with opaque water, which is conceptually divided into four quadrants and placed in a room with various visual cues on the walls and ceilings. A 10x10cm transparent hidden platform is located in the center of the target quadrant (Quandrant-3) 0.5 to 1.0 cm below the water surface. Mice were trained to locate the hidden platform from different starting points over 4 trials/block, 2 blocks/day, for 5 consecutive days. The position of the animal was tracked with an automated Ethovision video system (Noldus Information Technology, Leesburg, VA). For each trial, the mouse was allowed to search for the platform for up to 60 seconds, and the time to reach the platform was recorded. Afterwards it was placed on the platform to rest and learn the location for 10 seconds between trials. 24 hours after the final trial, the mice were tested in a probe test in which the platform was removed, so the animals could explore the maze freely for 60 seconds. The time, distance, and platform crossings in each quadrant were recorded. 

*Open Field Assay (OFA*) Mice were transferred to the testing room at a light level of 480 lux and a sound level of 60db generated by a white noise generator. The animals were allowed to acclimate to room conditions for 30 minutes. To start the test, a mouse was placed in the center of a clear Plexiglas chamber (40cm x 30cm x 30cm). Their movements were monitored by photo beams for 30 minutes using a Versamax animal activity monitor system (AccuScan Instruments, Columbus, Ohio). 

*Conditioned Fear (CF*) The test was performed as we previously described [10]. Briefly, in the CF paradigm, baseline freezing was recorded before any sound or foot shock stimuli on the first day (pre-shock freezing), and then an electric foot-shock was paired with an auditory cue. On the second day, the mice were first tested for their response to the exact same test chamber in which they received the foot-shock (contextual freezing). Afterwards, the environmental settings of the test chamber were drastically altered and the mice were placed back in the modified context. Their freezing frequency was first recorded without any auditory cue to establish a baseline, which will be subtracted in final calculation, and then the auditory cue was presented to test the fear response to the cue (cued freezing). All the freezing frequency was automatically recorded and calculated by the FreezeFrame software (San Diego Instruments, San Diego, CA). 

*Hotplate nociception test (HP*) Mice were acclimated to the testing room for 30 minutes and the hotplate was pre-warmed to 55°C during this time. To start the test, a mouse was placed in the center of the hotplate. The time before the mouse showed its first hindlimb response (jumping, shaking, or licking) was recorded as a measurement of its pain sensitivity levels.

## Results

### Physiological expression of APP, PS1 and wildtype human tau in APP/PS1/htau mouse model

 Both APP and PS1 carrying FAD mutations in the APP/PS1/htau model were created using knock-in methods previously described [5,8], and their native promoters are not affected, ensuring the physiological levels of protein expression. As shown in Figure 1B&C, in contrast to AD transgenic models, the APP (22C11) and PS1 protein levels were comparable in APP/PS1 or APP/PS1/htau samples. The human-specific 6E10 antibody only recognizes full-length APP in APP/PS1 and APP/PS1/htau samples but not WT controls, confirming the existence of humanized Aβ. The wildtype genomic human *MAPT* transgene is under its native promoter control [9], and when compared to wildtype mouse tau, we only observed a mild overexpression (less than a 35% increase) of the human tau. Taken together, the APP/PS1/htau model is free of mutant APP and PS1 overexpression, and the wildtype human tau is also expressed at a physiologically relevant level. 

### The APP/PS1/htau Model Exhibits Tau Hyperphosphorylation

**Figure 2:**
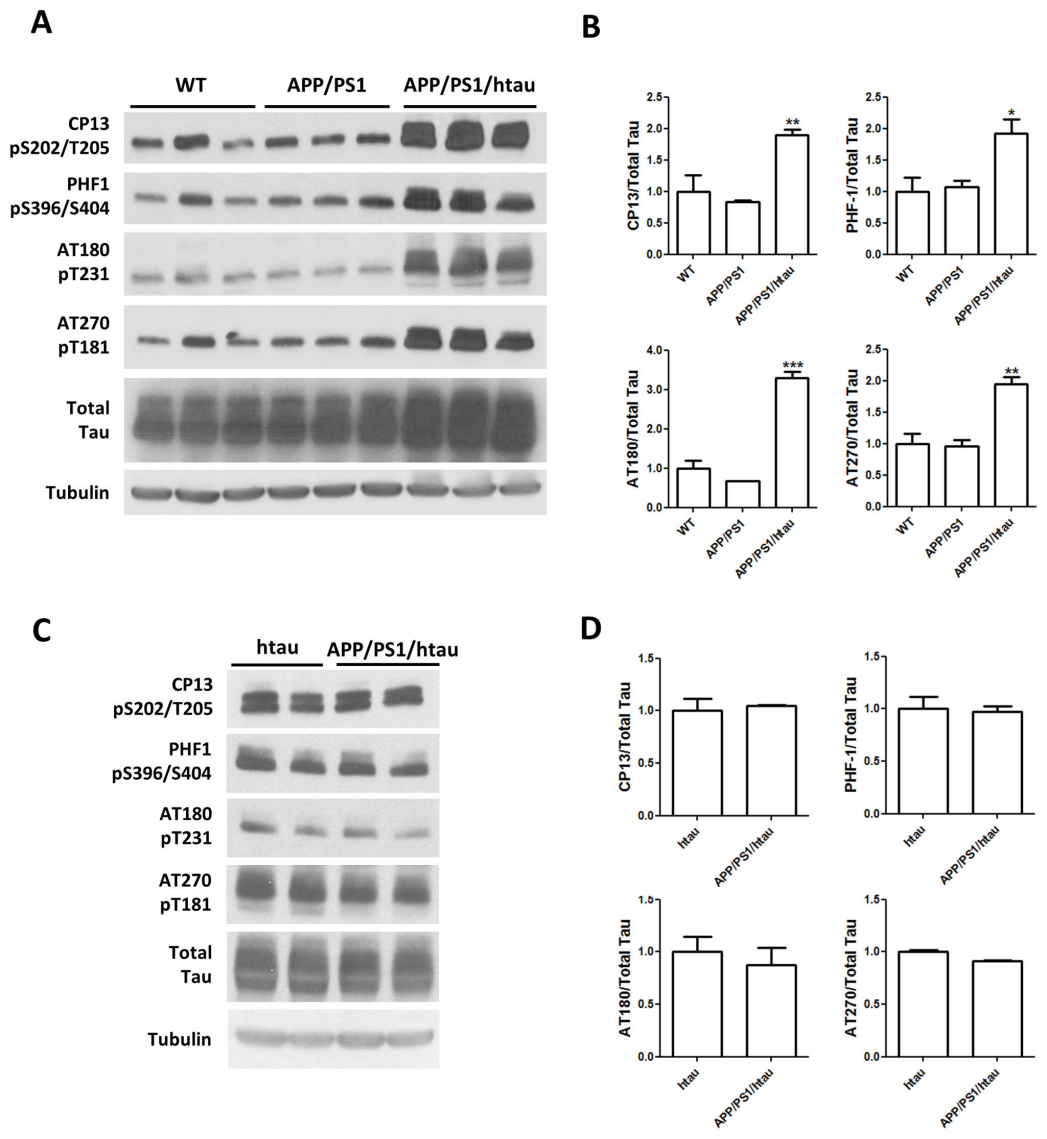
Biochemical analysis on tau phosphorylation in knock-in mouse models. A. Western blots using p-tau antibody CP13, PHF-1, AT180, AT270, and total tau antibody against hippocampal lysates from WT, APP/PS1 and APP/PS1/htau animals at 16 months of age. B. Statistics on band intensities of CP13/total tau, PHF-1/total tau, AT180/total tau, and AT270/total tau. * p<0.05, ** p<0.01, *** p<0.001, one-way ANOVA with Newman-Keuls multiple comparison test. C. Western blots using p-tau antibody CP13, PHF-1, AT180, AT270, and total tau antibody against hippocampal lysates from htau and APP/PS1/htau animals at 12 months of age. D. Statistics on band intensities of CP13/total tau, PHF-1/total tau, AT180/total tau, and AT270/total tau are shown below. Student t-test, no significant difference.

We first performed western blots using hippocampal lysates from WT, APP/PS1 and APP/PS1/htau animals at 16 months of age to quantitatively measure levels of tau phosphorylation at different sites. As shown in Figure 2A&B, compared to WT, there was no increase of phosphorylated mouse tau in APP/PS1 mice at all phosphorylation sites examined (pS202/pT205, pS396/pS404, pT231, and pT181). This result suggests that human Aβ may not augment mouse tau phosphorylation. However, after replacing mouse tau with the wildtype human tau, the APP/PS1/htau mice displayed tau hyperphosphorylation, reflected by significantly increased p-tau to total tau ratio despite a moderately increased total tau level (Figure 2A&B). Since htau mice alone have been reported to display increased tau phosphorylation [9], we next compared age-matched htau and APP/PS1/htau mice to determine if human Aβ could affect human tau phosphorylation in a mouse brain with the presence of APP and PS1 mutations. Interestingly, with or without APP/PS1, the human tau phosphorylation levels were similar at all sites examined (Figure 2C&D), suggesting that the tau hyperphosphorylation develops independently of Aβ accumulation in the APP/PS1/htau model.

**Figure 3:**
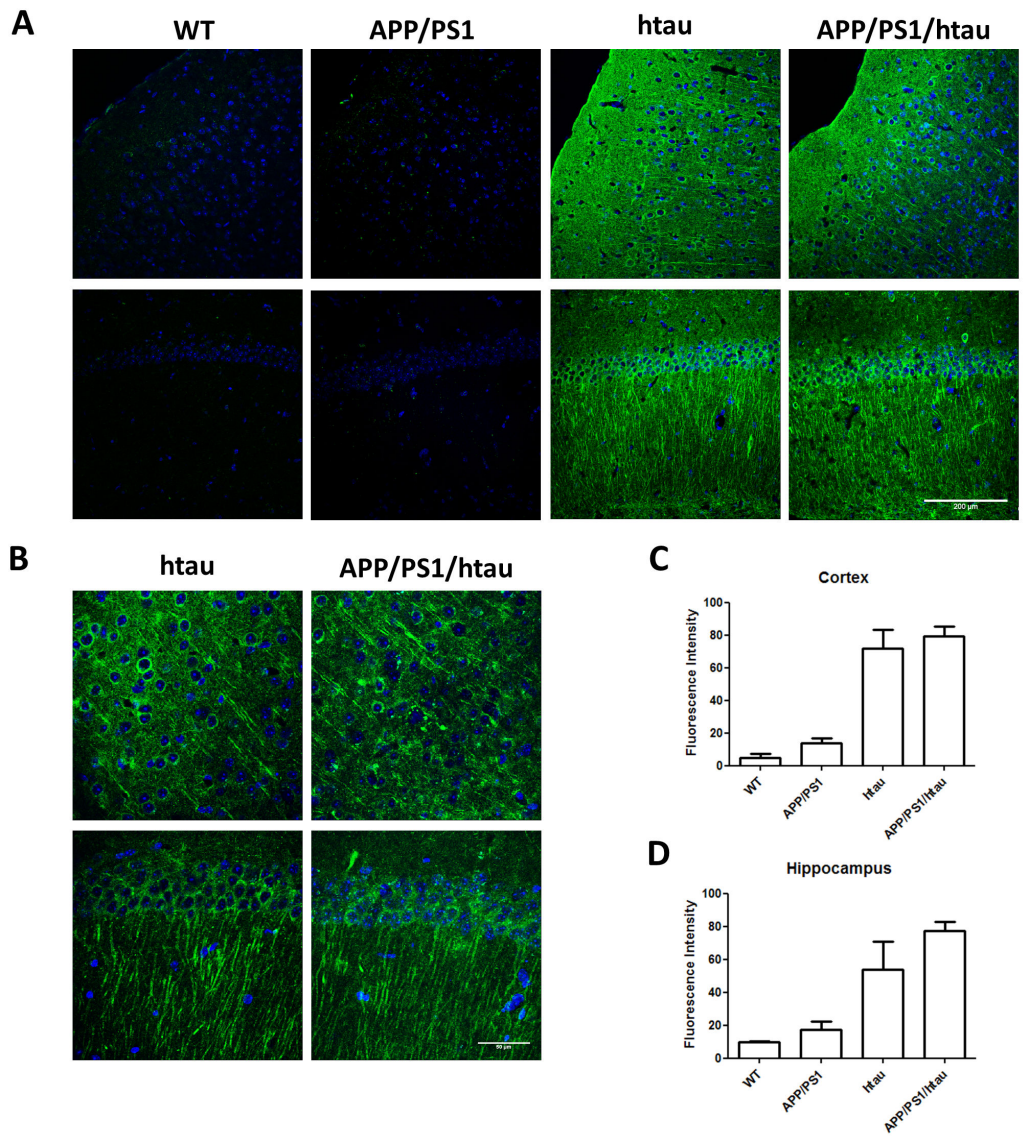
Immunofluorescence staining showing tau phosphorylation in the cortex and hippocampus of knock-in animals at 18 to 22 months of age. A. Frozen brain sections from WT, APP/PS1, htau, and APP/PS1/htau animals were stained against phosphorylated tau (CP13, green; DAPI, blue). Images were captured using 20X objective lens. Compared to WT, increased tau phosphorylation is present in htau and APP/PS1/htau animals, but not in APP/PS1 animals. Scale bar, 200µm. B. Images captured using 40X objective lens show structural details of phosphorylated tau (CP13, green; DAPI, blue) in htau, and APP/PS1/htau animals. Scale bar, 50µm. C&D. Quantification of fluorescence intensity of cortex and hippocampus from WT, APP/PS1, htau, and APP/PS1/htau brain sections.

To visualize p-tau localization in brain tissue, we performed immunofluorescence staining using CP13 antibody against WT, APP/PS1, htau, and APP/PS1/htau frozen brain sections at 18 to 22 months of age. As shown in Figure 3A, the levels of p-tau in WT and APP/PS1 animals remained minimal in both cortex and hippocampus, and APP/PS1 sections were indistinguishable from WT. In contrast, strong tau phosphorylation was detected in neuronal soma and dendrites in both htau and APP/PS1/htau sections (Figure 3A&B). Quantification of fluorescence intensity showed that tau phosphorylation levels were much higher in htau and APP/PS1/htau brains compared to the WT and APP/PS1 mice but they were similar to each other. Overall, p-tau western blot and immunofluorescence staining data are consistent, indicating APP/PS1 does not enhance tau phosphorylation in either mouse tau or wildtype human tau background up to about 20 months of age. 

### The APP/PS1/htau model develops Aβ deposition in advanced ages

**Figure 4:**
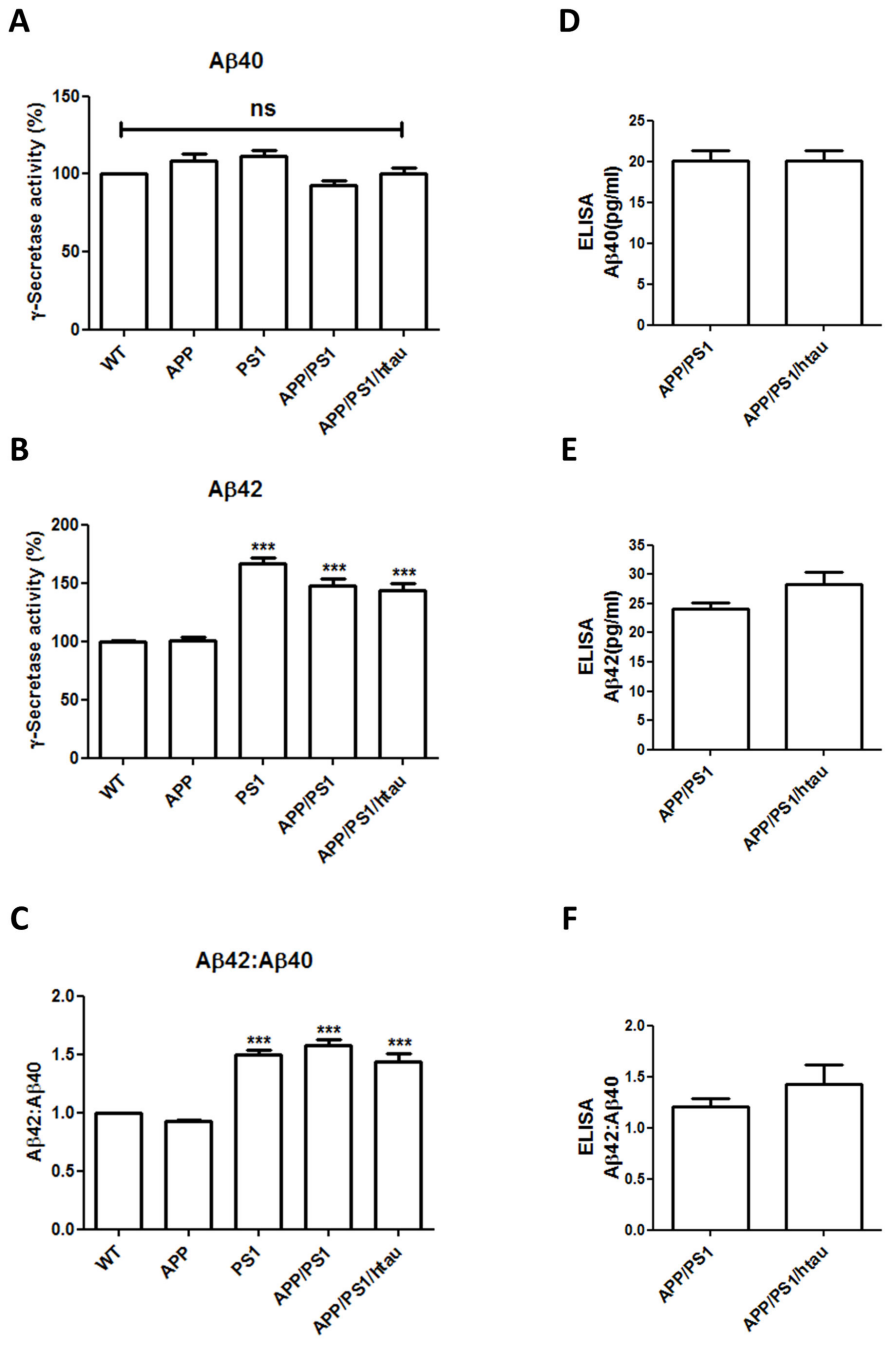
γ-secretase activity and Aβ levels are not affected by the presence of htau. A-C. Brain lysates from WT, APP, PS1, APP/PS1, and APP/PS1/htau animals at 3 to 4 months of age were analyzed using γ-secretase activity assay. PS1 M146V mutation does not alter Aβ40 but significantly increases Aβ42 production, leading to increased Aβ42:Aβ40 ratio in PS1, APP/PS1, and APP/PS1/htau samples. However, no significant difference in γ-secretase activity was found between PS1, APP/PS1, APP/PS1/htau samples. *** p<0.001, one-way ANOVA with Newman-Keuls multiple comparison test. D-F. Aβ ELISA was performed using brain lysates from APP/PS1 and APP/PS1/htau animals. Presence of htau does not change Aβ40 or Aβ42 production. Student t-test, no significant difference.

Our lab previously validated the presence of human Aβ in the APP knock-in mice [16], but the level is not sufficient to induce detectable plaque pathology within their lifespan (data not shown). We first examined the γ-secretase activity in WT, APP, PS1, APP/PS1, and APP/PS1/htau brain samples at 3 to 4 months of age. Aβ40 production remained unchanged across all the groups tested (Figure 4A). However, when compared to WT, Aβ42 production and Aβ42:Aβ40 ratio were significantly increased in the PS1, APP/PS1, and APP/PS1/htau samples but not in the APP sample (Figure 4B&C). Furthermore, there was no difference in Aβ42 production among all three genotypes that contain the PS1 M146V mutation, which is known to increase the production of highly fibrillogenic Aβ42 and reduce the secretion of Aβ40, leading to an increased Aβ42:Aβ40 ratio [12,17]. This result indicates that the observed γ-secretase activity change is dominated by the PS1 mutation, and neither APP mutations nor htau affects such change. We also measured the Aβ concentrations by ELISA in brain samples from APP/PS1 and APP/PS1/htau mice at 16 months of age to examine if increased tau phosphorylation affects the overall Aβ accumulation at a later age. Both Aβ40 and Aβ42 levels remained unchanged between the two groups (Figure 4D&E), as did the Aβ42:Aβ40 ratio (Figure 4F), suggesting that brain Aβ levels may not be affected by replacing mouse tau with human tau. 

**Figure 5:**
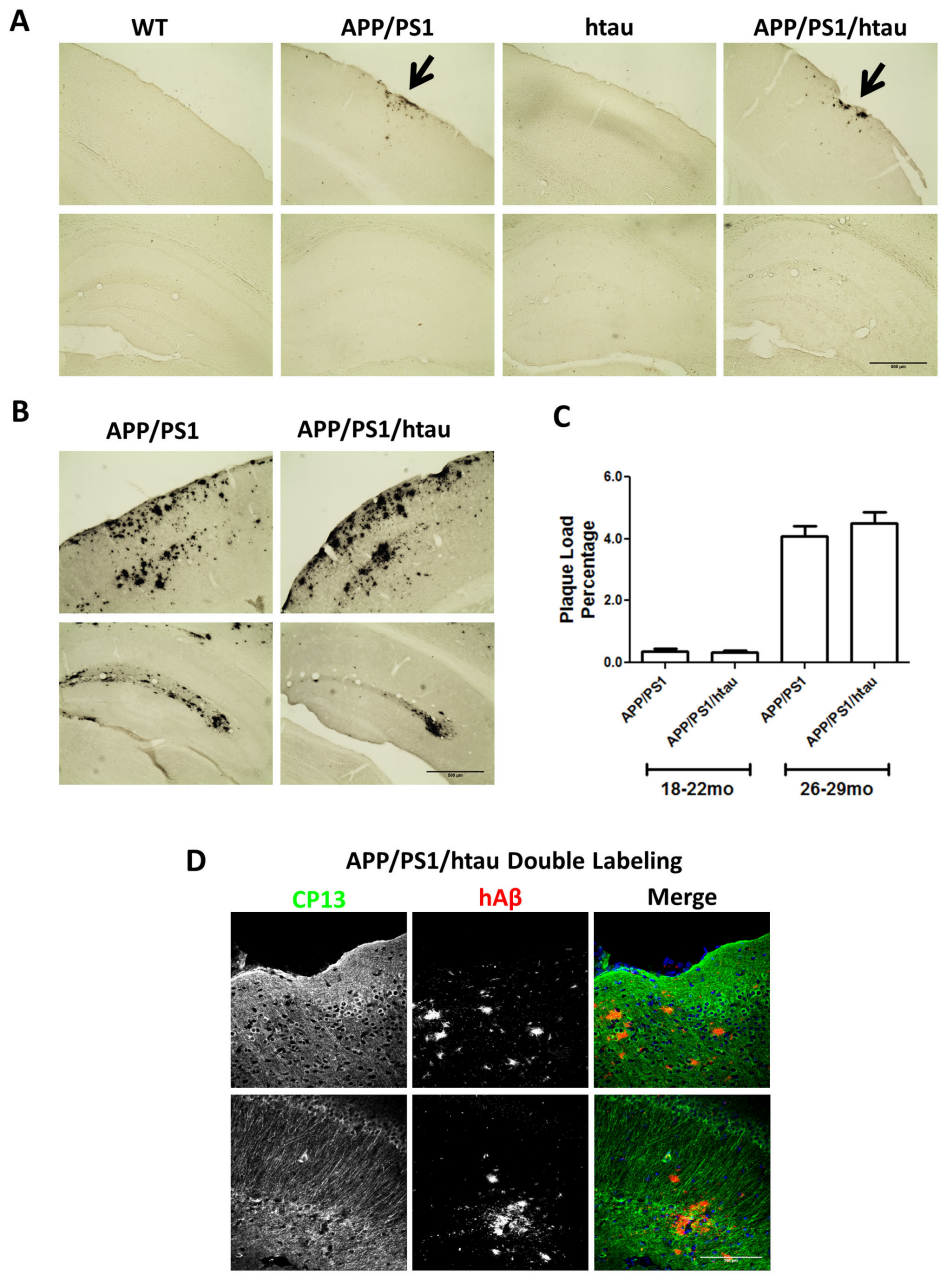
Plaque pathology increases with age in APP/PS1 and APP/PS1/htau animals. A. Immunohistochemical staining of 18-22 month-old WT, APP/PS1, htau, APP/PS1/htau brains. WT and htau brains are free of plaques. Scattered plaque deposition (black arrows) is detected in the outer layer of cortex in APP/PS1 and APP/PS1/htau brains, but not in hippocampus at this point. B. Immunohistochemical staining of 26-29 month-old APP/PS1 and APP/PS1/htau brains. Plaques have progressed into inner layers of cortex and become detectable in hippocampus. C. The brain plaque load in APP/PS1 and APP/PS1/htau samples at 18-22 and at 26-29 months of age. D. Aβ and p-tau double-labeling in APP/PS1/htau sections. Immunofluorescence staining of brain sections from 29 month-old APP/PS1/htau mice. Co-presence of p-tau (green) and Aβ plaque (red) with DAPI (blue) is shown in cortex, and hippocampus.

For plaque depositions, APP/PS1 and APP/PS1/htau animals showed obvious but mild plaque pathology when examined at 18-22 months (Figure 5A). Scattered Aβ plaques were readily detected on the outer edge of cortex but largely absent in hippocampus. No plaques were present in either WT or htau animals. The plaque pathology increased significantly in terminally aged APP/PS1 and APP/PS1/htau animals at 26-29 months. As shown in Figure 5B, prominent Aβ depositions progressed into inner layers of cortex and hippocampus. The plaque load increased significantly with age but remained comparable between APP/PS1 and APP/PS1/htau at both ages (Figure 5C). In addition, immunofluorescence staining using CP13 and human Aβ antibody against aged APP/PS1/htau cortex and hippocampus sections demonstrated the co-existence of the two key AD pathological hallmarks (Figure 5D). So far, this is the first AD mouse model that recapitulates both pathological hallmarks without overexpressing APP and PS1 or introducing tau mutations.

It was previously reported that tau deletion does not change Aβ levels in young transgenic AD mice [18,19]; here we compared brain samples of old APP/PS1/tau-/- and APP/PS1/htau at 18 months to further study the influence of tau on Aβ production. We have observed that neither γ-secretase activity nor BACE1 expression levels were changed by tau deletion (Figure S1 A&B). Consistent with this observation, APP/PS1/tau-/- and APP/PS1/htau mice displayed similar plaque pathology (Figure S1 C&D). Taken together with aforementioned tau phosphorylation studies, our data presented here indicates that Aβ pathology and tau hyperphosphorylation develop independently of each other.

### No overt learning and memory defects detected in APP/PS1/htau mice

**Figure 6:**
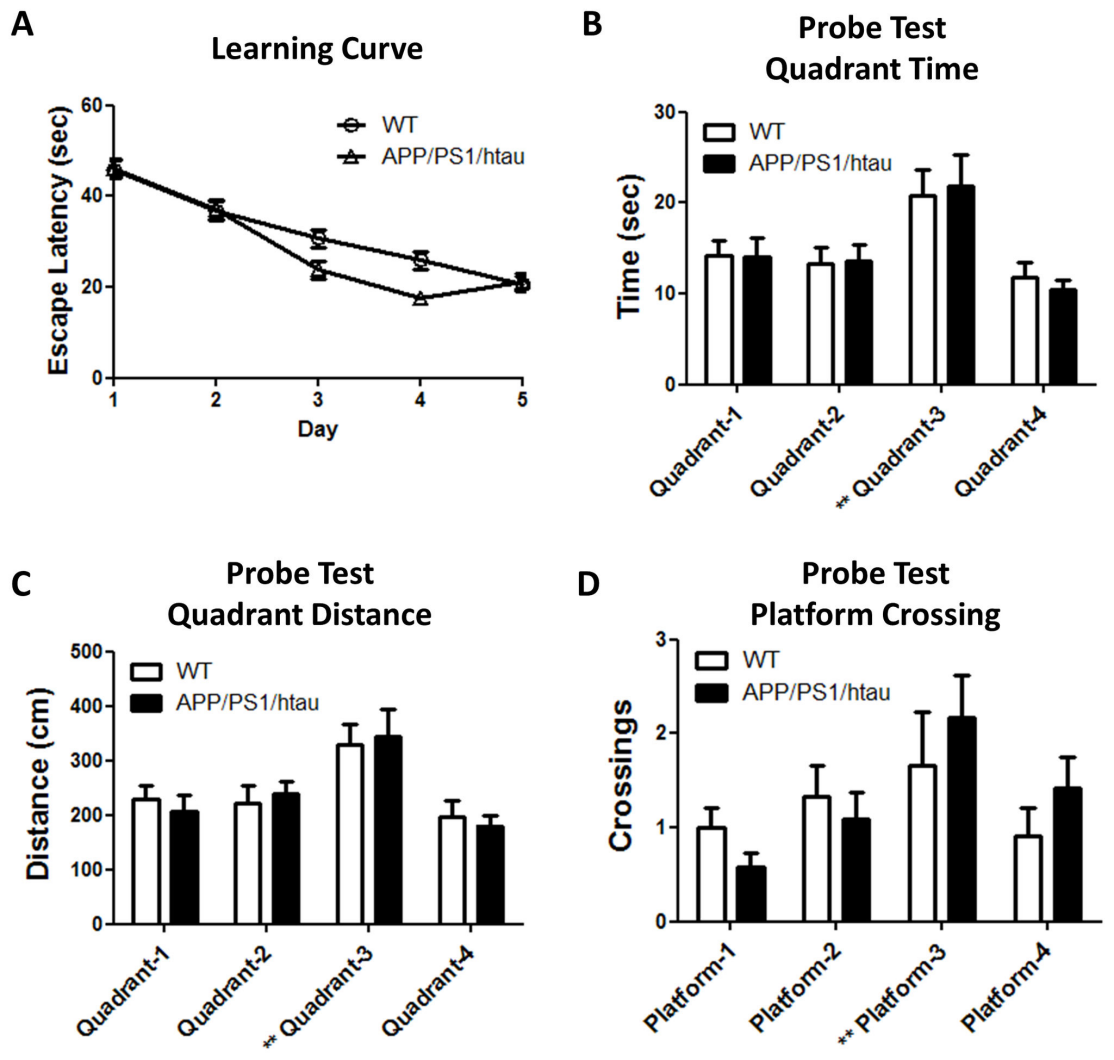
APP/PS1/htau animals did not show obvious learning and memory deficits in the Morris water maze test at 12 months of age. A. The learning curve. B-D. Probe test quantified with swimming time, swimming distance, and number of platform crossings. The target quadrant (Quadrant-3) is marked with **. N=12/group.

We performed the Morris water maze test, a well-accepted rodent learning and memory test, on WT and APP/PS1/htau animals at 12 months of age. The mice were trained for five consecutive days to learn the location of a hidden platform in the target quadrant (Quadrant-3), and then the platform was removed to evaluate the spatial memory of each mouse in the probe test. As shown in Figure 6A, the learning curves were calculated based on the time latency for a mouse to reach the platform. As the training continues, the mouse is expected to spend less time finding the platform. Compared to WT, the APP/PS1/htau group did not show appreciable learning impairment in locating the platform. Twenty-four hours after the initial test, the mice were re-introduced to the pool with the platform removed (probe test) to test if they displayed any quadrant preference. A mouse with a normal memory for the platform location is expected to spend more effort searching the nearby area; this is reflected by increased time spent, swim distance, and platform crossings at the original target quadrant. Both WT and APP/PS1/htau groups showed significantly increased searching activities in the target quadrant; however, there were no genotype differences (Figure 6B, C and D), indicating spatial memory was not overtly affected in the APP/PS1/htau mice at this age. 

### Increased anxiety and age-dependent reduced mobility

**Figure 7:**
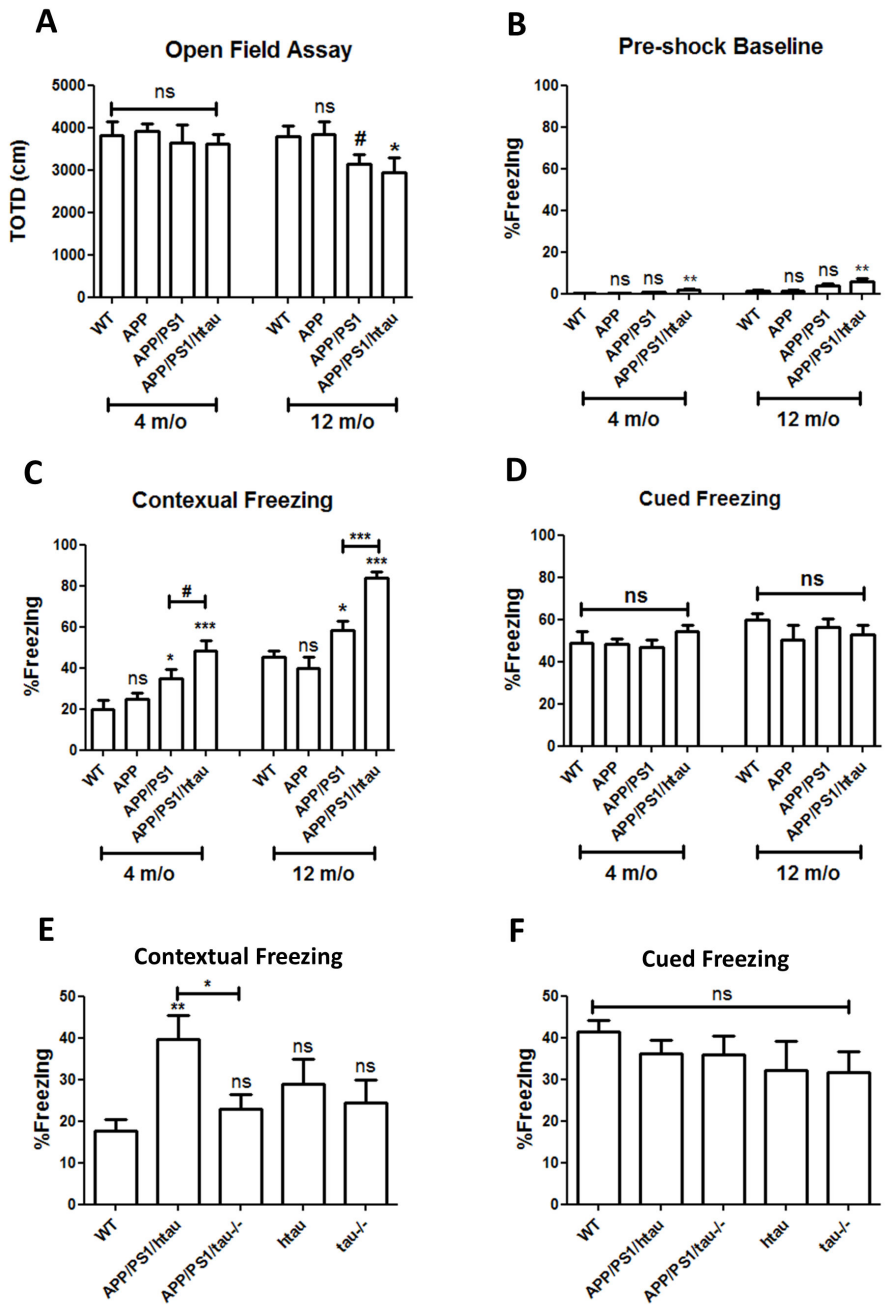
APP/PS1/htau animals showed age-dependent reduced activity and exaggerated fear response that can be ameliorated by tau deletion. A. The total travel distance (TOTD) was measured from WT, APP, APP/PS1 and APP/PS1/htau males at 4 and 12 months of age. At 4 months of age, there was no difference across different groups; but at 12 months of age, the locomotor activity was greatly reduced in APP/PS1 and APP/PS1/htau animals. # p< 0.06, *p<0.05, student t-test. B. Pre-shock baseline was measured immediately after the animals were put into the test chamber for the first time before any sound or shock stimuli. C. Contextual freezing. There was no difference between the WT and APP group, but the freezing frequency was significantly increased in the APP/PS1 group and further increased in the APP/PS1/htau group at both 4 and 12 months of age. D. Cued freezing frequency was calculated by subtracting the post-shock baseline from the observed freezing to the sound. There was no significant difference across all groups at both ages. *p<0.05, **p<0.01, ***p<0.001, student t-test. N=10-18/group. E. Contextual freezing of an independent cohort of WT, APP/PS1/htau, APP/PS1/tau-/-, htau, and tau-/- animals at a young age was measured. Compared to APP/PS1/htau animals, tau deletion in APP/PS1/tau-/- mice significantly reduced the freezing frequency to the WT level. htau and tau-/- animals show no significant change in freezing frequency. F. Cued freezing frequency remains the same across all the groups tested. *p<0.05, **p<0.01, student t-test. N=9-13/group.

We previously reported an increased anxiety-related phenotype of the APP and APP/PS1 mouse models due to central corticotrophin-releasing factor system perturbation [10]. In the present study, we also found similar levels of increased anxiety in APP/PS1/htau mice (Figure S2A). To ensure the enhanced anxiety-related stress does not affect the mobility of animals and thus further confound subsequent behavior analysis, we used the open field assay to evaluate their general locomotor activity. As shown in Figure 7A, at 4 months of age there was no difference in animal activity quantified by total travel distance in the test chamber in WT, APP, APP/PS1 and APP/PS1/htau mice, suggesting unaffected locomotor activity at this age. However, although the total travel distance in APP and WT animals remained comparable at 12 months, there was a trend of reduction (p<0.06) in APP/PS1 animals and it was significantly reduced in APP/PS1/htau animals (p<0.05). This indicates that the mutant APP or Aβ is not sufficient to cause the deteriorated movement performance but rather requires aging and the presence of the mutant PS1 and/or htau. 

### The APP/PS1/htau model exhibits exaggerated fear response, which can be ameliorated by tau reduction

The conditioned fear (CF) paradigm is used to test the ability of mice to associate an unpleasant foot-shock with the surrounding environment and sound when they experienced such shock. We previously observed increased freezing frequency in APP/PS1 mice [10]. To determine whether this phenotype is affected by htau addition, we performed the CF test on APP/PS1/htau mice. 

For the contextual freezing frequency, compared to the WT controls, the APP group did not show any changes, whereas the APP/PS1 and APP/PS1/htau mice displayed significantly increased freezing frequency at both 4 and 12 months of age. The APP/PS1/htau mice also showed a much higher freezing frequency than the age-matched APP/PS1 group (Figure 7C). Although we recorded slightly higher pre-shock freezing frequency in the APP/PS1/htau group (Figure 7B), this difference was not sufficient to cause the strong shock-induced freezing difference observed. On the other hand, the cued freezing frequency was largely unaffected across all the groups (Figure 7D). 

Previous studies demonstrated that knocking out tau in APP transgenic animals is beneficial, as tau deletion ameliorates the early lethality, seizure frequency, and behavioral deficits [18,19]. Here, we tested the hypothesis that tau deletion may reduce the exaggerated fear response. As shown in Figure 7E, we tested another cohort of animals in the CF paradigm to dissect the role of tau. As expected, the contextual freezing frequency of APP/PS1/htau group is much higher than WT controls. In contrast, the APP/PS1 mice on mouse tau knockout background (APP/PS1/tau-/-) is indistinguishable from WT, indicating tau deletion can almost completely block the increased freezing. The htau and tau-/- group do not show a significant difference with the WT group, excluding the possibility that tau on its own may affect the freezing response. More importantly, taking Figure 7C and 7E together, the results indicate that htau and APP/PS1 contribute to the freezing phenotype synergistically, and tau deletion not only removed the synergistic effect of htau but also blocked the effect of APP/PS1. The cued freezing frequency remained at similar levels across all groups tested (Figure 7F).

The increased freezing frequency of APP/PS1 and APP/PS1/htau mice is not likely due to pain threshold difference, as no difference was detected in nociception by the hotplate assay in all genotypes of mice tested above (Figure S2B). This further strengthens our conclusion that the observed freezing difference is caused by distinct intrinsic fear responses. 

## Discussion

In this study, we report the successful creation of the APP/PS1/htau AD mouse model that develops plaque deposition and tau hyperphosphorylation by expressing wildtype human tau and humanized Aβ without the overexpression of mutant APP or PS1. This mouse is, to our knowledge, closest to human AD genetics and pathophysiology. It is also an attractive *in vivo* system to study the interplay between Aβ and tau, to dissect their influence on animal behaviors, and to gain in-depth understanding on mechanisms of AD pathogenesis. 

The mechanisms linking Aβ and tau pathologies remain poorly understood. According to the prevailing Aβ cascade hypothesis, excessive amount of Aβ peptides generated by mis-regulated APP metabolism is considered to be the initiating factor of AD pathogenesis, leading to Aβ plaque formation, tau hyperphosphorylation, and neurodegeneration [20]. This hypothesis is directly supported by AD genetics [21], but has yet gained full support from AD mouse model studies. Although induction of tau hyperphosphorylation by Aβ has been reported in some studies, Roberson et al. found that the APP transgenic line J20 with aggressive Aβ pathology failed to show increased overall tau phosphorylation levels [18]. A recent study also reported that Aβ and tau hyperphosphorylation coexisted but in an independent manner in a double transgenic mouse model of human mutant APP (APP23) and wildtype tau (ALZ17) [22]. The authors noted that the mutant transgenic tau was used in previous publications that reported positive Aβ/tau interactions [23-26], thus questioning the relevance with human AD. 

Taking advantage of our APP/PS1/htau model, which resembles human AD genetics, we performed comprehensive biochemical and histological analyses to further study the interaction between Aβ and tau. We have found that p-tau levels are comparable between age-matched WT and APP/PS1 brain samples, suggesting APP/PS1 may not augment phosphorylation of wildtype mouse tau. Similarly, p-tau levels are also comparable between age-matched htau and APP/PS1/htau brain samples, suggesting APP/PS1 may not increase phosphorylation of wildtype human tau either. In addition, by comparing APP/PS1/htau with APP/PS1 or APP/PS1/tau-/- mice, we conclude that changes of p-tau levels or total tau levels does not affect γ-secretase activity, BACE1 levels, or plaque load, indicating that tau may not influence Aβ. Taken together, Aβ pathology and tau hyperphosphorylation are both present in the APP/PS1/htau model (Figure 5D), but appear to develop independently of each other. 

How do we reconcile the discrepancy between human AD and mouse models? One likely scenario is that some critical factors or modifiers may be absent in mice, which could be responsible for the apparent disconnection between the two pathological hallmarks. For example, it is well-accepted that neuroinflammation plays an important role in human AD pathogenesis for both Aβ pathology and tau hyperphosphorylation [27-31], which is also supported by mouse model studies that artificially induce brain inflammation [32-34]. Also, levels of pro-inflammatory cytokines, such as TNFα and IL-6, are reported to be increased with aging in human [35,36]. We evaluated levels of a number of key inflammatory cytokines, including TNFα, IL-1β, and IL-6, in brain samples from aged WT and APP/PS1/htau mice, and we also examined activated microglia and astrocytes in brain sections. However, no signs of overtly increased neuroinflammation were found in APP/PS1/htau samples (data not shown). Other possible missing factors may include oxidative stress, vascular risk factors, hyperglycemia, blood-brain barrier dysfunction, and possibly intrinsic differences between human aging and mouse aging. The APP/PS1/htau mice serve as a good model to search for the critical modifiers that link Aβ and tau, and these factors are likely key therapeutic targets for AD. 

Although the Aβ pathology and tau hyperphosphorylation seem to develop independently, they exhibit cooperative effects in behavior. By comparing APP/PS1/htau mice to their parental lines, we are able to dissect the contribution of each genetic factor to the observed behavioral abnormalities. While the APP single knock-in allele is sufficient to cause elevated anxiety-related behavior [10], the enhanced fear response requires the presence of both APP and PS1 knock-in alleles. Further, while htau itself does not have an effect on the freezing frequency, it contributes to the fear response in a synergic manner with APP and PS1. More interestingly, tau deletion can completely reverse this phenotype (Figure 7C&E). In contrast to the biochemical and pathological data above, this clearly shows the interaction of Aβ and tau in affecting higher brain functions. 

It is notable that the APP/PS1 mice exhibit enhanced fear response at 4 months of age, which is before any plaque deposition, indicating that this phenotype precedes and is most likely independent of plaque formation. Nevertheless, the increased amount of more toxic Aβ42 peptides conferred by the PS1 mutant allele may cause more profound neuronal activity changes in the APP/PS1 animals at early stages than APP alone. Furthermore, this enhanced fear phenotype was significantly augmented in APP/PS1/htau animals by introducing htau, and could be ameliorated by tau deletion. Although the exact physiological functions of tau are still under investigation, it is well accepted that tau plays an important role in regulating neuronal network activity; tau reduction was reported to have protective effects in APP transgenic mice and epilepsy animal models, rescuing seizure frequency, mortality, learning and memory deficits, and hyperactivity [18,19,37]. The underlying mechanism responsible for this cooperative effect of Aβ and tau is not clear at this time, but the new finding provides further support for targeting tau as a potential therapeutic approach in treating AD and possibly relevant mood disorders. 

APP/PS1/htau mice do not display any obvious impairment in the spatial learning and memory in a standard version of water maze compared to age-matched controls at 12 months (Figure 6). The negative result in one particular cognition behavioral test does not rule out the possibility that the APP/PS1/htau mice may show impaired performance in other types of tests or at different ages. We were not able to report water maze data in mice of older ages due to increased individual variability with aging. The reduced locomotor activity detected in 12 month-old APP/PS1/htau mice compared to WT controls (Figure 7A) further confound the interpretation of behavioral paradigms requiring locomotor activity such as water maze. Taken together, further studies are needed to fully evaluate the cognitive performance and other possible behavioral changes in aged APP/PS1/htau mice. 

In summary, the APP/PS1/htau model expresses target proteins at physiological levels and represents a novel humanized mouse model that faithfully reflects AD genetics. Both Aβ deposition and tau hyperphosphorylation are successfully recapitulated. Although no strong biochemical interactions between Aβ and tau have been identified despite comprehensive analysis, our studies support a synergistic interaction of Aβ and tau in brain function, in particular in fear response. Our findings presented here highlight both cooperative and independent effects of Aβ and tau, and provide valuable insights for modeling AD in mouse. 

## Supporting Information

Figure S1 **Influence of tau deletion on Aβ.** A. *in* *vitro* γ-secretase activity assay of brain samples from WT, APP/PS1/tau-/- and APP/PS1/htau mice at 18 months. Aβ40 production is comparable between all groups. Although much higher than WT, there is no significant difference of Aβ42 production between the APP/PS1/tau-/- and APP/PS1/htau samples. B. Western blot showing the expression levels of APP and BACE are comparable between APP/PS1/tau-/- and APP/PS1/htau samples. C. Representative images of plaque deposition in APP/PS1/tau-/- and APP/PS1/htau brains at 18 months. D. Brain plaque load quantification.
(TIF)

Figure S2 **Elevated Plus Maze (EPM) and hotplate assays.** A. At 4 months of age, all the APP, APP/PS1 and APP/PS1/htau mice spent significantly less time than the WT on the open arm of EPM. *p<0.05, student t-test, n=13-23/group. B. Hotplate assay. The latency to hindlimb response is comparable across all groups tested at 4 months. n=7-16/group.
(TIF)
