# Supplementary figures and images for: Modeling Alzheimer’s Disease in Mouse without Mutant Protein Overexpression: Cooperative and Independent Effects of Aβ and Tau
Source: PLoS One. 2013 Nov 20;8(11):e80706. doi: 10.1371/journal.pone.0080706 (PMC3835479; doi:10.1371/journal.pone.0080706)

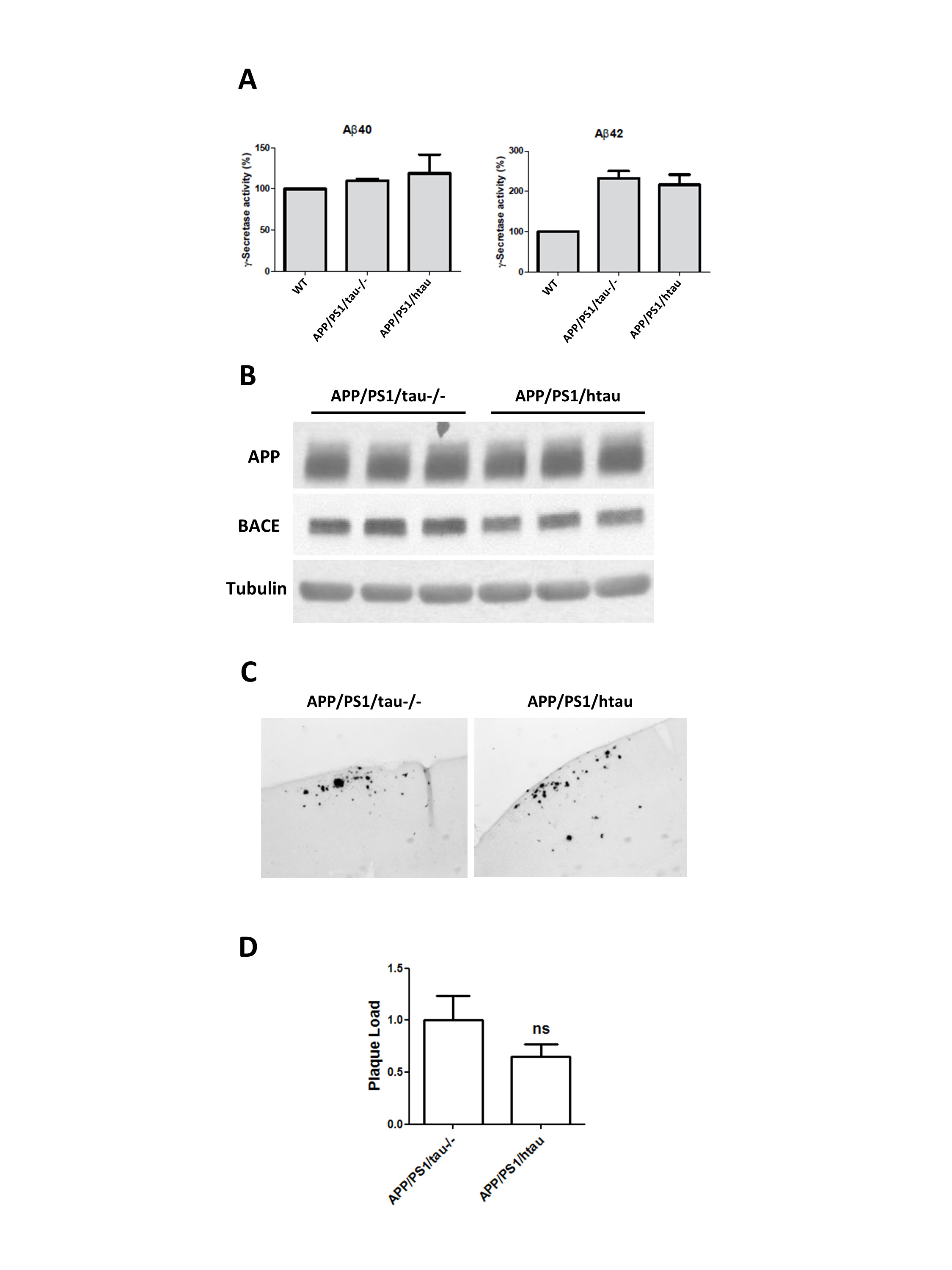

Supplement: Figure S1 — Influence of tau deletion on Aβ. A. in vitro γ-secretase activity assay of brain samples from WT, APP/PS1/tau-/- and APP/PS1/htau mice at 18 months. Aβ40 production is comparable between all groups. Although much higher than WT, there is no significant difference of Aβ42 production between the APP/PS1/tau-/- and APP/PS1/htau samples. B. Western blot showing the expression levels of APP and BACE are comparable between APP/PS1/tau-/- and APP/PS1/htau samples. C. Representative images of plaque deposition in APP/PS1/tau-/- and APP/PS1/htau brains at 18 months. D. Brain plaque load quantification. (TIF) [file pone.0080706.s001.tif]

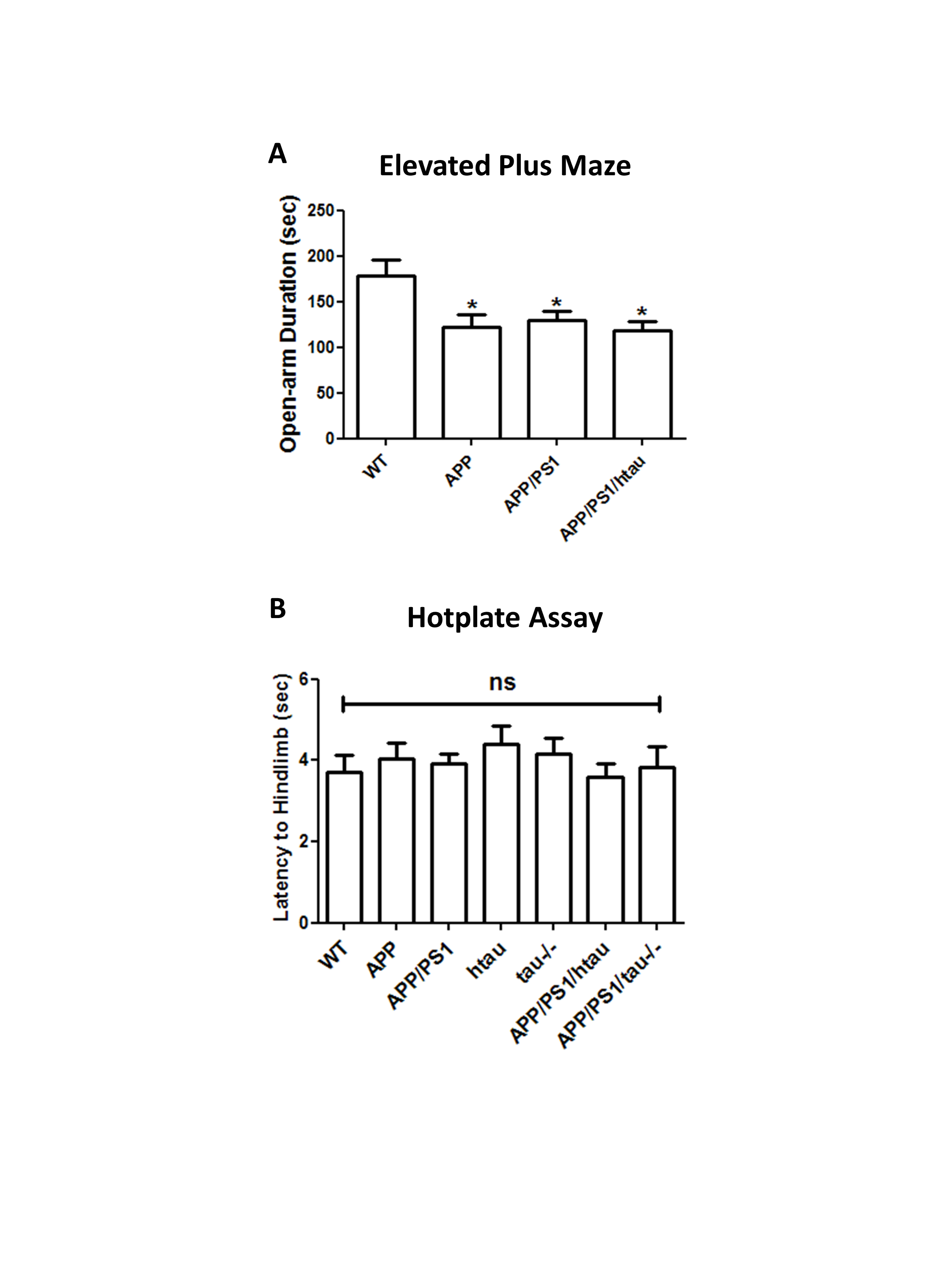

Supplement: Figure S2 — Elevated Plus Maze (EPM) and hotplate assays. A. At 4 months of age, all the APP, APP/PS1 and APP/PS1/htau mice spent significantly less time than the WT on the open arm of EPM. *p<0.05, student t-test, n=13-23/group. B. Hotplate assay. The latency to hindlimb response is comparable across all groups tested at 4 months. n=7-16/group. (TIF) [file pone.0080706.s002.tif]
